# Assisted Reproductive Technologies in Latin America: the Latin American Registry, 2021

**DOI:** 10.5935/1518-0557.20240107

**Published:** 2025

**Authors:** Fernando Zegers-Hochschild, Javier A. Crosby, Carolina Musri, Fanny Petermann-Rocha, Gustavo Martinez, Hitomi Nakagawa, Carlos Morente, Armando Roque, Ana Palma-Govea, on behalf of the Latin American Network of Assisted Reproduction

**Affiliations:** 1 Program of Ethics and Public Policies in Human Reproduction, Faculty de Medicine, University Diego Portales, Santiago, Chile; 2 Unit of Reproductive Medicine, Clínica Las Condes, Santiago, Chile; 3 SG Fertility Chile, Chile; 4 Medicina Reproductiva Fertilis, San Isidro, Buenos Aires, and Universidad de Belgrano, Buenos Aires, Argentina; 5 GENESIS, Centro de Assistência em Reprodução Humana, Brasília, Brazil; 6 Centro Medico PROAR, Rosario, Argentina; 7 Centro Especializado de Atencion a la Mujer (CEPAM), Hacienda de las Palmas, Huixquilucan, Estado de Mexico, Mexico; 8 IVI Panama, Ciudad de Panama, Panama; 9 Latin American Network of Assisted Reproduction (REDLARA), Montevideo, Uruguay

**Keywords:** ART Registry, embryo aneuploidy, oocyte donation, preimplantation genetic testing, reproductive outcome

## Abstract

**Research question:**

What are the trends and impact of new technologies on the effectiveness and safety of assisted reproductive technology (ART) performed in Latin America during 2021?

**Design:**

This was a retrospective collection of cyclebased multinational data obtained from ART procedures performed by 204 accredited institutions in 16 countries.

**Results:**

In total 127,351 initiated cycles resulted in 20,032 deliveries and 22,708 births. ART utilization showed great variability, from 623.5 cycles/million inhabitants in Uruguay to fewer than 35 in Guatemala and El Salvador. The proportion of women aged ≥40 years increased to 35.8%, while that of women ≤34 years dropped to 23.9%. Nonetheless, the proportion of single-embryo transfers (SET) increased from 11.9% in the previous decade to 42.4% in 2021. Of 22,708 babies born, 76.8% were singletons, 22.3% twins and 1.0% triplets or more. Intracytoplasmic sperm injection represented 84.5% of fertilization techniques, and blastocyst transfer increased from 49.6% in 2016 to 79.3% in 2021. The delivery rate after fresh blastocyst elective SET was significantly higher than after the transfer of one frozen embryo from a freezeall cycle (*p*<0.0001). The number of aspirations leading to preimplantation genetic testing has increased 2.8 times in 5 years and significantly increased delivery rates/transfer at all ages, including in oocyte donation (*p*≤0.002), and reduced miscarriage in women ≥35 years old. In oocyte donation, delivery rates after the fresh transfer of embryos from vitrified-warmed oocyte cycles generated similar outcomes to frozen embryo transfer. Perinatal mortality increased from 7.7 ‰ in singletons to 21.3 ‰ in twins.

**Conclusions:**

The systematic collection of cycle-based multinational data contributes to cooperative sustained development and helps implement evidence-based reproductive decisions.

## INTRODUCTION

This is the 33rd report of the Latin American Registry of Assisted Reproduction (RLA). Since 2012, reports have been published simultaneously in *Reproductive Bio-Medicine Online* (RBMO) and in *JBRA Assisted Reproduction*, the official journals of the Latin American Network of Assisted Reproduction (REDLARA). All publications starting in 1990 can be found at https://redlara.com/registro.asp.

The biomedical data presented here have been obtained via a cycle-based multinational registry providing detailed information on the utilization, availability, effectiveness, safety and perinatal outcomes of assisted reproductive technology (ART) treatments initiated between 1 January and 31 December 2021 with babies born up to September 2022. When relevant, longitudinal analyses were used to examine the trends over the previous decades. This report provides some additional information on the relative impact on ART outcome of preimplantation genetic testing (PGT), the influence of the number of eggs retrieved and the number of blastocysts generated.

## MATERIALS AND METHODS

Data on ART were collected from 204 centres in 16 countries in Latin America (Supplementary Table 1), covering the following: fresh autologous cycles of IVF and intracytoplasmic sperm injection (ICSI); PGT; frozen embryo transfer (FET) preceded by both fresh embryo transfer cycles and freeze-all cycles; oocyte donation, including the transfer of fresh and frozen-thawed embryos; fertility preservation; and embryo transfer cycles of embryos developed from vitrified-warmed oocytes (VWO), both autologous and heterologous.

All institutions reporting to the RLA have been accredited by an independent body within REDLARA. The forms used for this process can be obtained at www.redlara.com. Participating centres agree to have their data published by RLA. Therefore, no specific consent forms were requested for the scientific disclosure of data. The method of data collection in 2021 resembles that of previous years (Zegers-Hochschild *et al*., 2020), making the results comparable. The definitions used are those published in the International Glossary on Infertility and Fertility Care (Zegers-Hochschild *et al*., 2017). When calculating the clinical pregnancy rate (CPR) or delivery rate per oocyte retrieval, cases resulting in total embryo freezing were not included in the calculation. Furthermore, in the calculation of delivery rates, clinical pregnancies that were lost to follow-up were excluded. Historically, the rate of loss to follow-up was 3-8%, but during the COVID-19 pandemic this inadvertently increased to 10-12%, which impacted the previous calculation of delivery rates.

The cumulative delivery rate was calculated as previously described (Zegers-Hochschild *et al*., 2020) from aspirations and their related fresh and frozen transfer cycles taking place between January and December 2021. In this reporting year, cumulative deliveries were calculated from 181 institutions in 14 countries. Results were expressed as: (i) the cumulative delivery rate starting with all fresh transfers; and (ii) cumulative deliveries including only women having surplus frozen embryos apart from their fresh transfers.

### Statistical analyses

To test for the effect of age, the number of embryos transferred and the stage of embryo development at transfer on the delivery rate per embryo transfer, Poisson regression models with robust standard errors were used when analysing cross-sectional associations. The results are reported as prevalence ratios with 95% confidence intervals (95% CI). Poisson regression models with robust standard errors were used because they provide prevalence ratio estimates that are relatively easy to interpret, instead of odds ratios (Grant, 2014). Robust standard errors were used to correct underinflation when applying the Poisson model for binary outcomes. When variables were not stratified by age, analyses were adjusted for it. *p<*0.05 was considered statistically significant. Stata 18 statistical software (StataCorp LP, USA) was used to perform all the analyses.

## RESULTS

A total of 204 centres in 16 countries reported 127,351 initiated cycles (31% more than 2020), which resulted in 20,032 deliveries, 22,708 births and adding the estimated births in non-reporting institutions, a total of 25,116 births can be estimated in the region (Table 1).

**Table 1:** Assisted reproduction techniques reported in Latin America, 2021.

Country	Centers	FP	FRESH	FET (own)	OD	VWO	Total	Deliveries registered by RLA	Total number of births registered by RLA	Estimated total number of live births from ART	Estimated proportion live births from ART/ total births in the country
**Argentina**	21	1,953	8,768	4,737	5,293	628	21,379	3,057	3,246	3,340	0.63
**Bolivia**	3	15	299	48	251	29	642	137	161	251	0.11
**Brazil**	69	6,480	25,331	18,323	3,729	2,454	56,317	7,824	8,872	9,418	0.35
**Chile**	11	831	3,816	2,693	1,094	580	9,014	1,404	1,489	1,784	1.00
**Colombia**	14	258	1,395	828	853	153	3,487	638	777	847	0.14
**Costa Rica**	1	13	67	23	10	0	113	15	15	40	0.07
**Ecuador**	8	93	536	327	275	20	1,251	251	277	332	0.13
**El Salvador**	1	10	29	8	15	3	65	8	11	25	0.03
**Guatemala**	2	11	187	120	129	5	452	103	110	140	0.19
**Mexico**	47	1,012	8,651	5,496	5,628	426	21,213	4,259	5,169	5,873	0.31
**Panama**	4	89	541	365	195	50	1,240	263	282	341	0.51
**Paraguay**	1	29	176	153	84	15	457	48	56	115	0.10
**Peru**	15	1,430	3,281	1,845	2,319	759	9,634	1,558	1,727	1,792	0.56
**Rep. Dominicana**	2	14	147	62	103	2	328	99	113	224	0.14
**Uruguay**	2	110	635	507	353	31	1,636	347	379	495	1.43
**Venezuela**	3	2	57	39	22	3	123	21	24	98	0.02
**Total (%)**	**204**	**12,350 (9.7)**	**53,916 (42.3)**	**35,574 (27.9)**	**20,353 (16.0)**	**5,158 (4.1)**	**127,351**	**20,032**	**22,708**	**25,116**	**0.32**

FP, fertility preservation; FRESH, initiated fresh autologous IVF/ICSI cycles; FET, autologous frozen embryo transfer; OD, oocyte donation with fresh and frozen-thawed embryos; VWO, embryo transfer cycles with embryos developed from autologous and donated vitrified/warmed oocytes.

The important rise in the number of centres reporting to the RLA, with the concomitant rise in initiated cycles and babies born, was seen throughout most countries as they returned to normal functioning after the pandemic. Brazil remains the largest contributor, with 44.2% of all initiated cycles, followed by Argentina and Mexico with 16.8% and 16.7% cycles, respectively. In autologous reproduction, fresh IVF and ICSI cycles represented 42.3% of initiated cycles, followed by 27.9% of FET (25% in 2020). Oocyte donation cycles remained high (16%) compared with approximately 7.6% in European countries (European IVF Monitoring Consortium (EIM) for the European Society of Human Reproduction and Embryology (ESHRE), 2023). This high proportion of oocyte donation cycles is consistent with the increasing proportion of women aged ≥40 years.

**Figure 1:**
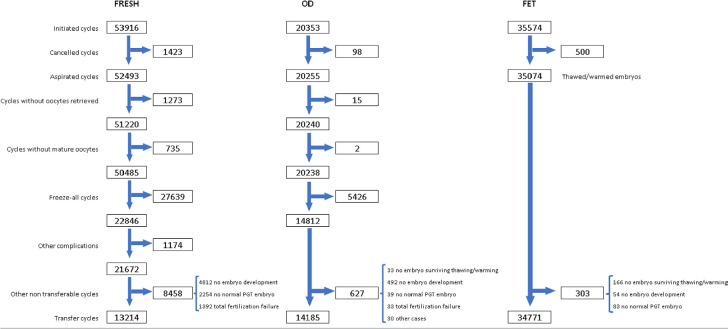
Events that affect the outcome of Fresh in vitro fertilization and intracytoplasmic sperm injection (IVF/ICSI), fresh and frozen oocyte donation (OD) and autologous frozen embryo transfer (FET) in Latin American ART Registry, 2021. PGT, preimplantation genetic testing (PGT-A, PGT-M, PGT-SR reported together).

The sequence of events that need to be considered when looking at a specific outcome can be found in Figure 1, starting with initiated cycles and cancellations before follicle aspiration; then aspirations with or without mature oocytes, freeze-all oocytes, embryos or both; the number of cycles with fertilized oocytes or failed fertilization; and the number of cycles with viable embryos for transfer or normal embryos after PGT. After all these events have been considered and adjusted, the CPR and delivery rates can be calculated, and comparisons made. This detailed description, however, is only possible in a cycle-based data collection system.

### Utilization of ART in Latin America

Utilization of ART is expressed as the total number of ART cycles performed per million inhabitants. Considering that not all cycles carried out in every country were reported to the Latin American registry, the best possible estimate of the non-reported cycles was obtained through information provided by regional directors of REDLARA, embryologists, clinicians and industry representatives. The magnitude of the estimates, which constitutes a potential source of error, was expressed as degrees of confidence according to Dyer and colleagues (Dyer *et al*., 2019) and later applied by Zegers-Hochschild and collaborators (Zegers-Hochschild *et al*., 2021).

**Figure 2:**
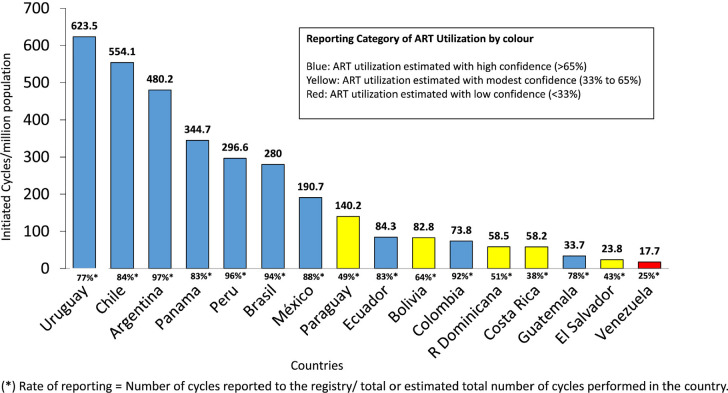
Utilization of assisted reproductive technology. Estimated number of initiated cycles per million inhabitants by country in Latin American ART Registry, 2021.

As seen in Figure 2, the RLA collects data on a vast proportion of ART cycles carried out in most countries in the region, and covers between 88% and 97% of the major contributors. Uruguay, with a law providing universal care and a well-established state funding programme, has the highest utilization rate (623.5 cycles/million), followed by Chile, which increased from 372 cycles/million in 2019 to 554.1 cycles/million in 2021 by incorporating a state programme covering half of a fixed price established by the government. Utilization in Argentina, despite having a law providing universal care, dropped from 490 cycles/ million in 2019 to 480.2 cycles/million in 2021, mainly due to economic limitations. These moving trends in utilization reflect how access to ART is affected, especially in countries where most of the funding is out of pocket.

### Age of the women and number of embryos transferred

**Figure 3:**
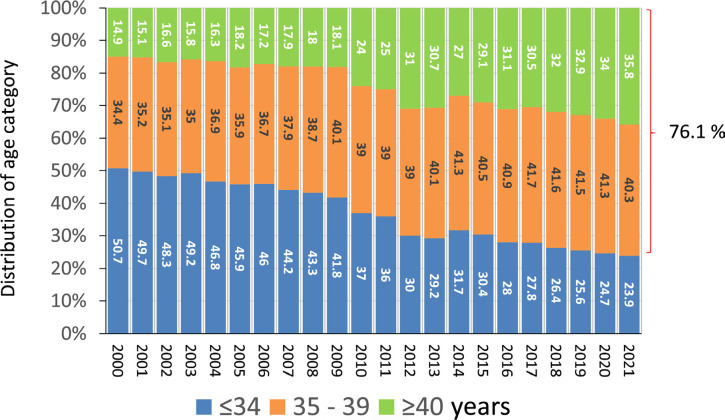
Age distribution of female partner in Fresh in vitro fertilization and intracytoplasmic sperm injection (IVF/ICSI) cycles in Latin American ART Registry, 2000-2021.

As seen in Figure 3, in the past two decades, the proportion of women aged ≤34 years dropped from 50.7% to 23.9%, while that of women aged ≥40 increased from 14.9% to 35.8%. The rising age of women requesting ART constitutes a global phenomenon and what is experienced in Latin America is also experienced in Europe among other regions. However, in Europe the proportion of women aged ≥40 years is only 25.5% and women ≤34 years of age represent 43.8% (European IVF Monitoring Consortium (EIM) for the European Society of Human Reproduction and Embryology (ESHRE), 2023). This increasing proportion of women aged ≥40 years in Latin America is one of the reasons for the rise in the number of oocyte donation cycles.

**Figure 4:**
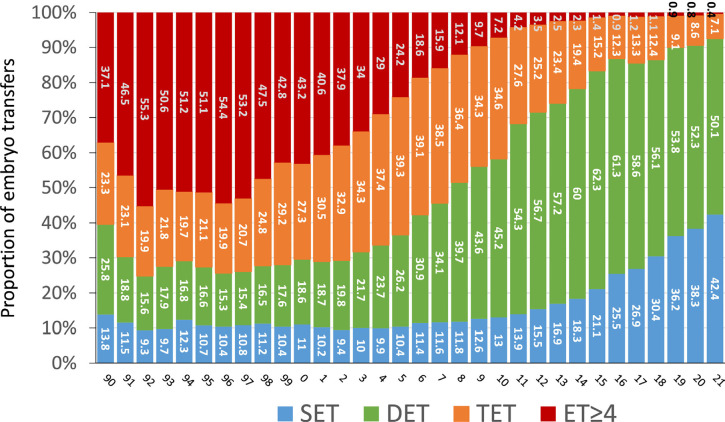
Number of embryos transferred in Fresh autologous transfers in the last 32 years in Latin American ART Registry 1990 - 2021. SET: single embryo transfer; DET: double embryo transfer; TET: three embryos transferred; ET≥4: four or more embryos transferred.

However, in spite of an increasing proportion of reproductively older women, the mean number of embryos transferred dropped from 3.2 in the year 2000 to 1.7 in 2021 (Figure 4). Furthermore, in the last two decades, the proportion of single-embryo transfers (SET) increased from 11% in 2000 to 42.4% in 2021 (38.3% in 2020), while the proportion of transfers involving three or more embryos dropped from 70.5% in 2000 to 7.5 in 2021. In 2021, 92.5% of all fresh transfers included a maximum of two embryos.

### Outcome of autologous fresh IVF and ICSI cycles according to the women’s age and number of embryos transferred

In 2021, there were 53,916 fresh initiated IVF/ICSI cycles and, consistent with previous years, the proportion of ICSI remained very high (84.5%). After discarding freezeall cycles and other conditions resulting in no embryos for transfer, the number of transfer cycles dropped to 13,214 (see Figure 1).

**Figure 5:**
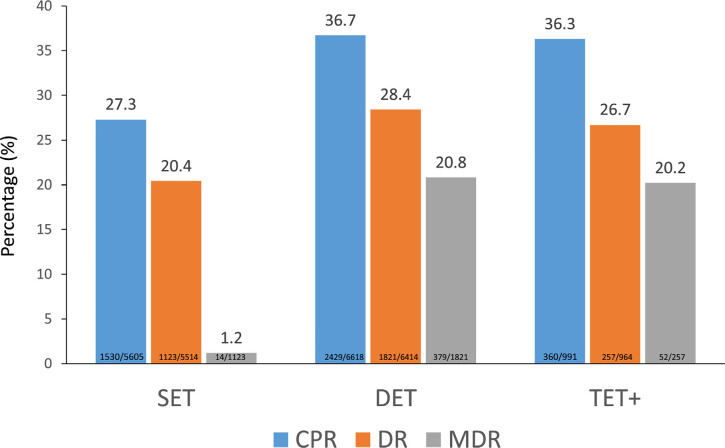
Clinical pregnancy rate (CPR), delivery rate (DR) and multiple delivery rate (MDR) per embryo transfer in autologous fresh IVF and ICSI cycles according to the number of embryos transferred in Latin American ART Registry, 2021. SET: single embryo transfer. DET: double embryo transfer: TET+: triple or more embryo transfer.

**Figure 6:**
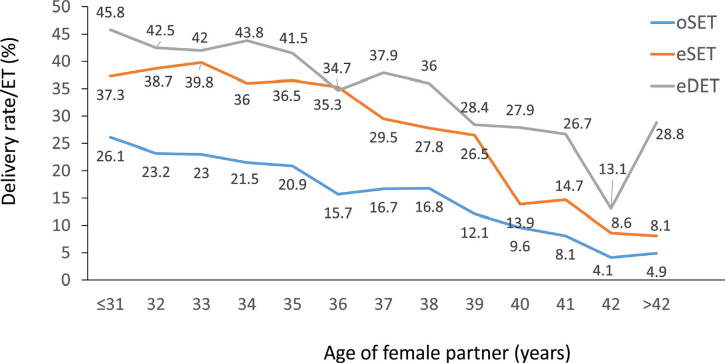
Delivery rate per embryo transfer (DR/ET) in autologous fresh IVF and ICSI cycles according to the age of the female partner and the number of embryos transferred in Latin American ART Registry, 2021. eSET: elective single-embryo transfers; oSET: transfer of only one embryo because there are no more embryos available for transfer; eDET: elective double-embryo transfers.

The CPR and delivery rate per oocyte retrieval and embryo transfer according to the women’s age are shown in Table 2. Because of the high prevalence of ICSI procedures, the results of ICSI and IVF have been combined. As expected, the chances of birth were affected by the number of embryos transferred (Figure 5) and age of the female partner (Figure 6). Both the CPR and delivery rates were significantly higher when transferring two compared with one embryo (both *p<*0.0001). Furthermore, transferring three embryos (see Figure 5) did not further increase the number of pregnancies or deliveries; its major impact was in terms of multiple births, which rose from 1.2% after SET to 20.8% and 20.2% in double-embryo transfer (DET) and triple-embryo transfer, respectively. The major difference was the higher proportion of triplets after triple-embryo transfer (5.8% of multiples) compared with 0.8% after DET.

**Table 2:** Clinical pregnancy rate and delivery rate in fresh autologous IVF and ICSI cycles stratified according to the age of women in 2021.

Age of women	Oocyte retrievals	Oocyte retrievals^*^	Oocyte retrievals^**^	Clinical Pregnancies (CPR/OR^*^)	Deliveries (DR/ OR^**^)	PR (95% CI); p-value	Embryo transfers^***^	Deliveries (DR/ET^***^)	PR (95% CI); p-value
<30	3,825	1336	1300	450 (33.7%)	352 (27.1%)	1.00 (Ref.)	954	352 (36.9%)	1.00 (Ref.)
31-33	5,941	2381	2322	756 (31.8%)	591 (25.5%)	0.94 (0.84; 1.05); 0.284	1,660	591 (35.6%)	0.96 (0.87; 1.07); 0.506
34-36	10,344	4429	4347	1,180 (26.6%)	928 (21.3%)	0.79 (0.71; 0.88); <0.001	2,905	928 (31.9%)	0.87 (0.78; 0.96); 0.004
37-39	13,787	6375	6287	1,171 (18.4%)	849 (13.5%)	0.50 (0.45; 0.56); <0.001	3,481	849 (24.4%)	0.66 (0.60; 0.73); <0.001
40-42	13,003	6857	6805	626 (9.1)	399 (5.9%)	0.22 (0.19; 0.25); <0.001	2,912	399 (13.7%)	0.37 (0.33; 0.42); <0.001
>43	5,593	3476	3471	136 (3.9%)	82 (2.4%)	0.09 (0.07; 0.11); <0.001	980	82 (8.4%)	0.23 (0.18; 0.28); <0.001
**Total**	**52,493**	**24,854**	**24,532**	**4,319 (17.4%)**	**3,201 (13.0%)**	**-**	**12,892**	**3,201 (24.8%)**	**-**

After comparing, DR shows no difference between IVF/ICSI we group them together to analyze only the effect of age.

* Oocyte retrievals (OR) excludes cases of total embryo freezing.

** OR excluding cases of total embryo freezing and pregnancies with loss to follow-up (LFU).

*** Em bryo transfers (ET) excluding pregnancies with LFU.

CPR/OR^*^: clinical pregnancy rate per oocyte retrieval; DR/OR^**^: delivery rate per oocyte retrieval excluding LFU; DR/ET^***^: delivery rate per transfer excluding LFU

### Outcome of autologous IVF and ICSI after elective and non-elective SET and DET

There were 5605 fresh SET and 6618 fresh DET cycles. Each of these was further stratified into elective SET (eSET; 41%) and elective DET (eDET; 41.5%). As seen in Table 3, which includes the transfer of both cleaving embryos and blastocysts, CPR and delivery rates were significantly greater after eSET than the transfer of only one embryo because there were no more embryos available for transfer (oSET) (38.9% and 30.3%, compared with 19.2% and 13.5%, respectively; prevalence ratio for clinical pregnancy: 1.75, 95% CI 1.60-1.91, *p<*0.001; prevalence ratio for delivery rate: 1.84, 95% CI 1.65-2.06, *p<*0.001). A similar and significant relationship was also established when comparing eDET and the transfer of only two embryos because there were no more embryos available for transfer (prevalence ratio clinical pregnancy: 1.44, 95% CI 1.35-1.54, *p<*0.001; prevalence ratio delivery rate: 1.46, 95% CI 1.35-1.58, *p<*0.001). Furthermore, when only blastocysts were transferred, the delivery rates were significantly higher than after the transfer of cleaving embryos (*p<*0.0001) in both eSET and eDET (Supplementary Figure 2).

**Table 3:** Clinical pregnancy rate, delivery rate and gestational order in elective and non-elective SET and DET in fresh autologous IVF/ICSI in 2021.

Type of transfer	Embryo transfers		Clinical pregnancies		Embryo transfers^*^	Deliveries							
	Number	%	Number	%	Number	Number of deliveries	Delivery rate per embryo transfer^*^ (%)	Singleton (n)	Singleton (%)	Twin (n)	Twin (%)	≥Triplets (n)	>Triplets (%)
oSET	3,309	59.0	636	19.2	3,262	440	13.5	437	99.3	3	0.7	0	0
eSET	2,296	41.0	894	38.9	2,252	683	30.3	672	98.4	10	1.5	1	0.1
oDET	3,872	58.5	1,149	29.7	3,766	850	22.6	696	81.9	154	18.1	0	0
eDET	2,746	41.5	1,280	46.6	2,648	971	36.7	746	76.8	222	22.9	3	0.3

* excluding cases of pregnancies with loss to follow-up (LFU).

eSET, elective single-embryo transfers; oSET, transfer of only one embryo because there are no more embryos available for transfer; eDET, elective doubleembryo transfers; oDET, the transfer of only two embryos because there are no more embryos available for transfer.

When examining the impact of elective and non-elective fresh transfers at different ages (Figure 6), the delivery rate after eSET was significantly higher than after oSET at all ages (*p=*0.0001); as expected, eDET values were also significantly higher than those after eSET (*p<*0.0001).

### Influence of blastocyst *versus* cleaving embryo transfer

**Figure 7:**
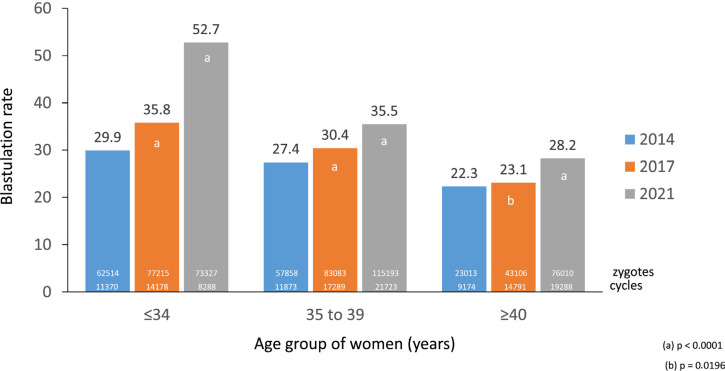
Blastulation rate (# transferred blastocysts + # vitrified blastocysts / # zygotes) according to women’s age group in Latin American ART Registry, in three years: 2014, 2017 and 2021.

**Supplementary Figure 1:**
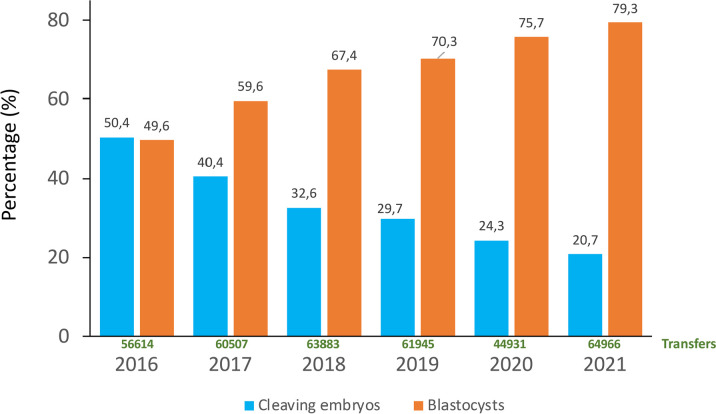
Proportion of transfers according to the stage of development: cleaving embryo or blastocyst transfers in all transfers from fresh autologous cycles of IVF and intracytoplasmic sperm injection, frozen embryo transfer cycles, oocyte donation cycles, including the transfer of fresh and frozen- thawed embryos and vitrified-warmed oocyte cycles including both autologous and heterologous cycles.

**Supplementary Figure 2:**
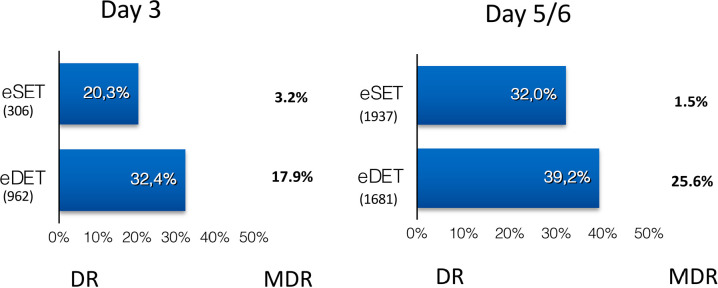
Delivery rate (DR) and multiple delivery rate (MDR) per embryo transfer in fresh autologous IVF and ICSI cycles according to eSET and eDET and the day of embryo transfer in Latin America, 2021. eSET: elective single-embryo transfers; eDET: elective double-embryo transfers.

The proportion of blastocyst transfers compared with cleaving embryos increased from 49.6% in 2016 to 79.3% in 2021 (Supplementary Figure 1). This has been possible thanks to major improvements in laboratory conditions supporting long term in-vitro culture. As seen in Figure 7, the blastulation rate, or the capacity of zygotes to reach the blastocyst stage (number of blastocysts transferred + blastocysts vitrified, divided by the number of zygotes generated), has improved in the last 7 years, reaching 52.7% in women aged ≤34 years, 35.5% in women 35-38 years old and 28.2% in women ≥40 years. Furthermore, when comparing the delivery rate and multiple births (as an indirect expression of implantation rate), after the elective transfer of 8-cell cleaving embryos (day 3 only) and elective transfer of blastocysts (Supplementary Figure 2), delivery rates were significantly higher after the transfer of blastocysts, in both eSET and eDET (delivery rate: both *p<*0.0001; multiple delivery rate: eSET, *p=*0.3175, eDET, *p=*0.0079). Furthermore, delivery rate/embryo transfer after blastocyst eSET is the same as after cleaving embryo eDET; however, the proportion of multiple births rises from 1.5% to 17.9%. This is evidence for a clear benefit from transferring one elective blastocyst rather than two elective day 3 cleaving embryos.

**Figure 8:**
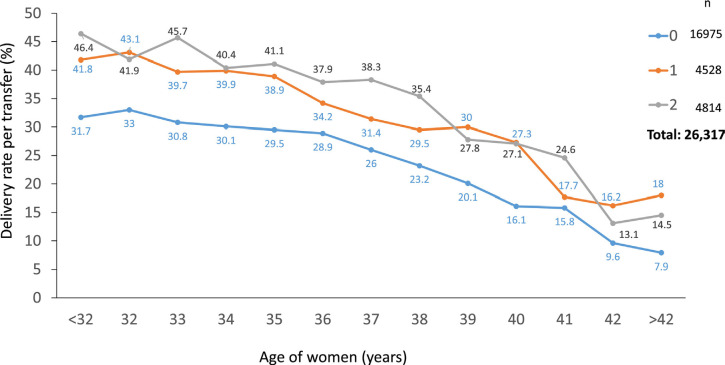
Delivery rate in Fresh transfers in the last 4 years of the Latin American ART Registry according to the age of women and the number of extra blastocysts vitrified in the same cohort. 0: no other blastocyst vitrified; 1: one extra blastocyst vitrified; 2: two extra blastocysts vitrified.

From another perspective, the number of blastocysts generated by a woman or couple has a direct impact on the chances of birth after a fresh transfer. As seen in Figure 8, which includes 26,317 blastocyst transfers over 4 years follow-up, the delivery rate after a fresh transfer is significantly higher in women having one or two extra blastocysts, compared with women having no blastocyst left after their fresh transfer (*p=*0.0001). The generation of three or more blastocysts does not seem to increase the chances of delivery after a fresh transfer but influences the cumulative chances of birth (data not shown here).

### FET cycles

**Figure 9:**
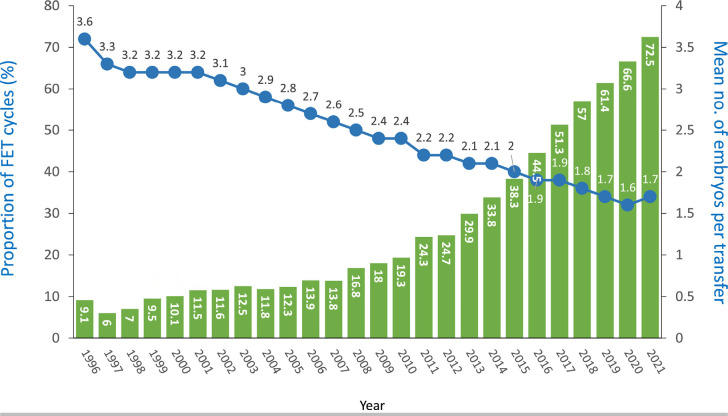
Proportion of frozen embryo transfer (FET) cycles and mean number of embryos per transfer in Latin American ART Registry, 1996-2021.

Out of 62,170 embryo transfer cycles, 44,916 were FET cycles (34,771 autologous + 10,145 oocyte donation; see Figure 1). The proportion of FET over fresh transfers in autologous cycles increased from 9.1% in 1996 to 72.5% in 2021 (Figure 9). The increasing use of this technology has contributed to a major decrease in the number of embryos transferred, from a mean of 3.2 to 1.7 embryos in only 20 years. Out of 15,702 autologous FET cycles, excluding PGT and freeze-all cycles (Table 4), the overall CPR, delivery rate and multiple births per transfer were 38.7%, 28.9% and 11.1%, respectively. This better outcomes in FET over fresh transfer - 32.7%, 24.8% and 13.9% (values extracted from Figure 5) - are observed with one and two embryos transferred (SET: *p<*0.0001; DET: *p<*0.0001). The better outcome of FET over fresh transfers is probably multifactorial, including better endometrial receptivity and a higher proportion of blastocyst transfer in FET (89.1%), compared with fresh transfers (55.6%) (data not shown).

**Table 4:** Clinical pregnancy rate, delivery rate and gestational order according to the number of embryos transferred in Autologous FET cycles. in 2021.

Number of embryos transferred	Embryo transfers^¥^		Clinical pregnancies		Embryo transfers^*^	Deliveries							
	Number	%	Number	%	Number	Number of deliveries	Delivery rate per embryo transfer^*^ (%)	Singleton (n)	Singleton (%)	Twin (n)	Twin (%)	>Triplets (n)	>Triplets (%)
1	8,415	53.6	2,951	35.1	8,151	2,114	25.9	2083	98.5	31	1.5	0	0.0
2	6,875	43.8	2,972	43.2	6,583	2,148	32.6	1708	79.5	429	20.0	11	0.5
>3	412	2.6	160	38.8	402	118	29.4	102	86.4	13	11.0	3	2.5
Total	15,702	100	6,083	38.7	15,136	4,380	28.9	3,893	88.9	473	10.8	14	0.3

¥ Excludes vitrified-warmed oocyte, PGT and Freeze all cycles

* Excluding cases of pregnancies with loss of follow-up (LFU).

### Freeze-all cycles

During 2021 there were 27,639 follicular aspirations leading to freeze-all autologous cycles (52.7% of all follicular aspirations; see Figure 1). On the other hand, there were 9987 autologous freeze-all transfer cycles resulting from procedures performed in 2021 and in previous years.

Freeze-all transfer cycles generated 4255 clinical pregnancies and 3024 deliveries, with an overall delivery rate per transfer of 31.8%, discarding cases with loss to follow-up (Table 5). The CPR and delivery rate were significantly higher after freeze-all cycles (42.6% and 31.8%, respectively; Table 5) compared with FET resulting from a previously failed fresh transfer (38.7% and 28.9%, respectively, Table 4; CPR: *p<*0.0001; delivery rate: *p<*0.0001). Nonetheless, when freeze-all cycles were compared with elective fresh transfers of only blastocysts (Supplementary Table 2), the delivery rate after fresh blastocyst transfer (32% after eSET and 39.2% after eDET) was significantly greater than after freeze-all cycles (Table 5; 26.8% in SET and 35.9% in DET; *p<*0.0001 and *p=*0.0160, respectively).

**Table 5:** Clinical pregnancy rate, delivery rate and gestational order according to the number of embryos transferred after Autologous Freeze all cycles, 2021.

Number of embryos transferred	Embryo transfers^¥^		Clinical pregnancies		Embryo transfers^*^	Deliveries							
	Number	°/o	Number	°/o	Number	Number of deliveries	Delivery rate per embryo transfer^*^ (%)	Singleton (n)	Singleton (%)	Twin (n)	Twin (%)	>Triplets (n)	>Triplets (%)
1	4,401	44.1	1,575	35.8	4,265	1,141	26.8	1,124	98.5	16	1.4	1	0.1
2	5,032	50.4	2,415	48.0	4,726	1,696	35.9	1,250	73.7	438	25.8	8	0.5
>3	554	5.5	265	47.8	530	187	35.3	124	66.3	61	32.6	2	1.1
Total	9,987	100	4,255	42.6	9,521	3,024	31.8	2,498	82.6	515	17.0	11	0.4

¥ Excludes cases with PGT.

* Excluding cases of pregnancies with loss of follow-up (LFU).

### Endometrial preparation for FET

Endometrial preparation for embryo transfer was compared between hormonal replacement *versus* ultrasound monitoring of a natural cycle. Out of 34,771 autologous FET, 4622 (13.3%) embryos were transferred in a monitored natural cycle and 30,149 (86.7%) after endometrial preparation with oral oestradiol and vaginal progesterone. The age distribution of women and the mean number of embryos transferred was similar in both groups. The mean age was 36.8 years in both groups and the mean number of embryo transfers was 1.44 (SD 0.542) in natural cycles and 1.45 (SD 0.567) in hormone replacement cycles (*p=*0.4324). The CPR and delivery rate were 42.5% (1964/4622) and 32.8% (1457/4444) in monitored natural cycles, and 42.7% (12887/30149) and 32.8% (9479/28912) in hormone replacement cycles. No differences were found between groups (CPR: *p=*0.7980; delivery rate: *p=*1.000).

### Influence of PGT on ART outcome

**Supplementary Figure 3:**
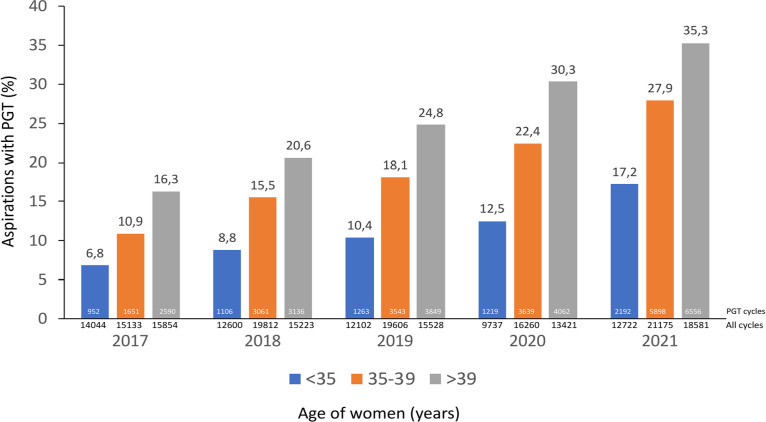
Five years trend on the use of PGT in different age groups in Latin America, 2017-2021.

In the last 5 years, the overall number of PGT cycles has increased 2.8 times, while the proportion of aspirations leading to PGT increased from 11.5% in 2017 to 27.9% in 2021(Supplementary Figure 3). In 2021, 173 out of 204 centres (84.8%) reported 14,646 aspirations leading to PGT. This corresponds to 29.0% of the 50,478 aspirations with at least one mature oocyte. When stratified by age, the percentage of aspirations associated with PGT was 18.1% in women aged ≤34 years, 30.6% in women aged 35-39 years and 31.9% in women ≥40 years. However, the largest increment since 2020 has been in women aged ≤34 years (3.1 times).

The effect of PGT on the delivery rate and miscarriage rate can be seen in Table 6. Excluding cases with loss to follow-up of clinical pregnancies, there were 6102 FET/PGT cycles, of which 5122 transfers were from autologous cycles (83.9%) and 980 (16.1%) from oocyte donation. The delivery rate per embryo transfer was significantly greater with PGT in all autologous age groups (all *p<*0.001) and in oocyte donation cycles (*p=*0.002) compared with those without PGT. Furthermore, with PGT, there were no differences in delivery rate/embryo transfer in autologous young (<35 years) and reproductively older (>39 years) women (*p=*0.1127). On the other hand, the miscarriage rate was significantly lower after PGT in women aged ≥35 years (*p<*0.001). No significant differences were found in younger women.

**Table 6:** Effect of PGT on the delivery rate and miscarriage rate according to age of women in autologous FET and OD FET (2021).

Outcome	Age of women	FET with PGT	FET without PGT	Prevalence Ratio (95% CI); *p*-value	
Delivery^*^	Oocyte Donors	38.3% (375/980)	33.4% (2,503/7,497)	0.87 (0.80; 0.95); 0.002^¥¥^	
	Autologous <35	43.3% (424/980)	36.4% (2,763/7,587)	0.84 (0.78; 0.91); <0.001^¥¥^	
	Autologous 35 - 39	43.0% (1,005/2,338)	29.8% (2,948/9,882)	0.69 (0.66; 0.73); <0.001^¥¥^	
	Autologous >39	40.2% (725/1,804)	21.8% (1,423/6,519)	0.54 (0.50; 0.58); <0.001^¥¥^	
Miscarriage^*^	Oocyte Donors	18.4% (85/461)	18.8% (588/3,121)	1.03 (0.83; 1.26); 0.809^¥^	
	Autologous <35	14.8% (74/500)	16.0% (531/3,320)	1.08 (0.86; 1.35); 0.498^¥^	
	Autologous 35 - 39	13.2% (155/1,171)	20.2% (751/3,720)	1.52 (1.30; 1.79); <0.001^¥^	
	Autologous >39	12.5% (105/837)	25.0% (482/1,929)	1.99 (1.64; 2.42); <0.001^¥^	

FET, frozen embryo transfer; OD FET = oocyte donation frozen embryo transfer; PGT, preimplantation genetic testing; Prevalence Ratio.

Oocyte donors were <35 years. All analyses were adjusted by women age. Clinical pregnancies with LFU were excluded.

(^*^) For miscarriage the denominator is clinical pregnancies, for deliveries, the denominator is embryo transfers.

¥ Likelihood of having a miscarriage. The reference group is “with PGT”.

¥¥ Likelihood of delivery. The reference group is “with PGT”.

Comparisons were also made between autologous pregnancies in young women (≤34 years) and pregnancies generated by oocyte donation, both following PGT. The mean age of the donors was 25.1 years. The delivery rate/embryo transfer in oocyte recipients (38.3%) was significantly lower compared with women aged ≤34 years (43.3%) (*p=*0.0244). Furthermore, the rate of miscarriage, although statistically not significant, appeared to be higher (18.4%) in oocyte recipients compared with women ≤34 years old (14.8%) (*p=*0.1335). This suggests that the age and health of the oocyte recipients influences the chances of carrying a viable clinical pregnancy.

### Frequency of aneuploidy in human embryos

**Figure 10:**
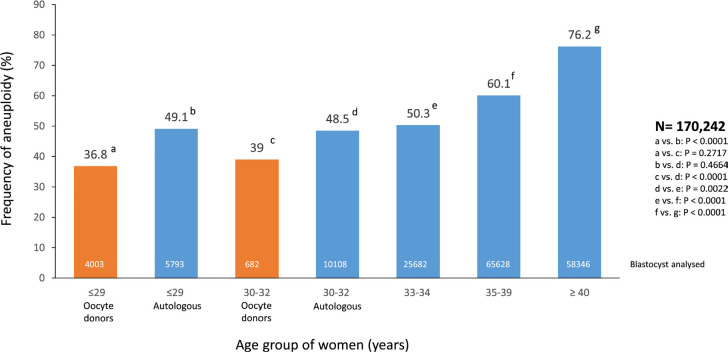
Frequency of aneuploidy in blastocysts according to the age of woman in the last five years in Latin American ART Registry, 2017-2021.

Between 2017 and 2021, a total of 170,242 blastocysts were examined for either aneuploidy or single-gene defects. The technique most frequently used in Latin America is next-generation sequencing. Figure 10 shows the frequency of aneuploidy in different age groups and in embryos generated by oocyte donors. As seen, there is a progressive and very significant rise in aneuploidy as women age, reaching 76.2% in women aged ≥40 years (33-34 *versus* 35-39 years: *p<*0.0001; 35-39 *versus* 40 years: *p<*0.0001). In addition, embryos generated by young infertile women had a higher chance of aneuploidy than embryos resulting from oocyte donors of a similar age (≤29 years and 30-32 years; *p<*0.0001 in both age groups). This finding suggests that the lower delivery rate/embryo transfer and higher miscarriage rate in oocyte recipients (Table 6) most likely results from pathologies attributable to the host rather than to the embryos.

### Outcome of oocyte donation cycles

**Figure 11:**
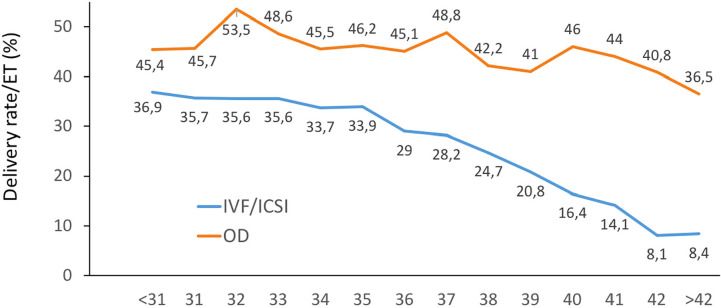
Delivery rate per embryo transfer (DR/ET) in fresh autologous IVF and ICSI and fresh oocyte donation (OD) cycles according to the age of the female partner in Latin American ART Registry, 2021.

As seen in Table 1, there were 20,353 initiated cycles representing 16.0% of all cycles performed in the region. After discarding cancellations, freeze-all cycles and other factors, there were 14,185 embryo transfers. In contrast with autologous reproduction, delivery rates in oocyte donation were practically unaffected by the age of the recipients (Figure 11). Furthermore, as shown in Table 6, the miscarriage rate after an FET in oocyte donation without PGT (18.8%) was much lower than what would be expected considering the age of the oocyte recipients (41.9 [SD 4.96] years).

The CPR, delivery rate and multiple birth rates were examined in 4040 fresh embryo transfers of embryos generated from fresh donated oocytes, 10,145 FET of embryos generated from fresh donated oocytes and 2727 transfers (fresh + FET) of embryos generated from donated VWO (Supplementary Tables 3-5, respectively). A summary analysis of these three treatment modalities in oocyte donation showed, first, higher delivery rates after fresh embryo transfer of embryos generated from fresh donated oocytes (41.1%) compared with FET (34.8%) of embryos generated from fresh donated oocytes or compared with fresh transfers and FET combined from embyros generated from donated VWO (35.3%) (*p<*0.0001; *p=*0.0043, respectively).

Second, delivery rates after the transfer of two embryos (fresh or frozen generated from fresh donated oocytes) were always higher than after the transfer of one embryo (fresh embryo transfer 44.4% *versus* 35.9%, *p<*0.0001; FET 38.6% *versus* 31.7%, *p<*0.0001). However, the proportion of multiple births increased more than 20 times after the transfer of two embryos (Supplementary Table 3: from 1.2% to 30.1%; Supplementary Table 4: from 1.2% to 25.3%).

Third, the transfer of three fresh embryos does not increase delivery rates (*p=*0.5043). However, the proportion of twins and triplets is approximately 40 times higher than after the transfer of one embryo (multiple delivery rate: from 1.2% to 45.2%; Supplementary Table 3).

Fourth, the delivery rate/embryo transfer after the transfer of fresh embryos developed from donated VWO is similar to that in FET cycles of embryos generated from fresh donated oocytes (38.2% *versus* 34.8%, *p=*0.7800). However, the transfer of frozen-thawed embryos generated from donated VWO significantly lowers the chances of clinical pregnancy and delivery when compared with the transfer of fresh embryos generated from donated VWO (41.2% *versus* 49.9%, *p=*0.0001; 26.4% *versus* 38.2%, *p=*0.0041, respectively).

### Cumulative delivery rate

**Figure 12:**
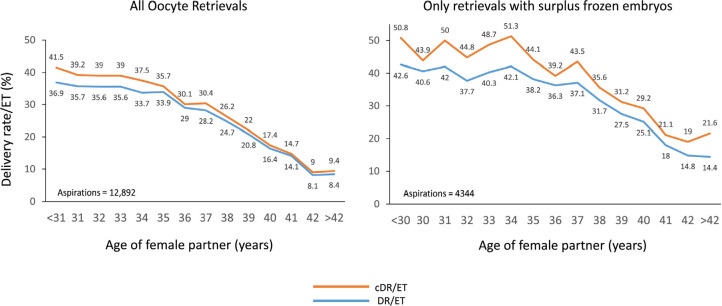
Cumulative delivery rate (cDR) per embryo transfer in IVF and ICSI cycles according to the age of the female partner in Latin American ART Registry, 2021. Left panel: all aspirations irrespective of whether there were frozen embryos for further transfer; right panel: only aspirations with surplus frozen embryos.

Cumulative delivery rates were calculated in a cohort of 12,892 women irrespective of whether they had surplus frozen embryos for delayed transfer, and in a subgroup of 4344 cases where all women had at least one extra embryo frozen for further transfer, irrespective of whether they were used during 2021. For the calculation of cumulative deliveries, this latter group is the one that better reflects what the cumulative chances are, since women who do not have frozen embryos do not have a cumulative chance of birth; their only chance is after the fresh transfer. As seen in Figure 12, the delivery rate per fresh transfer is already higher at all ages in women with surplus frozen embryos compared with the whole cohort of women (with and without surplus embryos), in which 66.3% of aspirations did not produce surplus embryos. An interesting observation in the sub-cohort of women with surplus frozen embryos was the less pronounced drop in delivery and cumulative delivery rates as age increases compared with the whole cohort.

### Perinatal outcome and preterm birth

**Figure 13:**
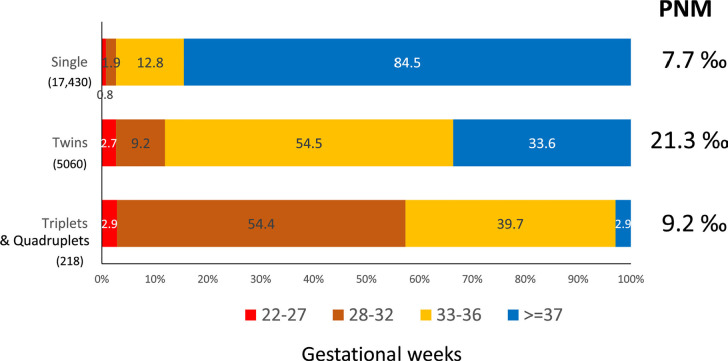
Preterm birth data for 20,032 deliveries and perinatal mortality (PNM) rates from 22,708 births according to order of gestation in Latin American ART Registry, 2021.

Perinatal mortality (PNM) and preterm births (Figure 13) were calculated from 20,032 deliveries and 22,708 births. Of these births, 17,430 (76.8%) were singletons, 5060 (22.3%) twins and 216 (1.0%) triplets or more. Consistent with previous years, PNM was 7.7‰ in singletons (134/17430), rising to 21.3 ‰ in twins (108/5060) and 9.2 ‰ in triplets and more (2/218). This last value is very low due to small number of cases, but historically PNM in triplets has been of the order of 60%. On the other hand, preterm birth was 15.5% in singletons, rising to 66.4% in twins and 97.1% in triplets. Of these, the percentage of extreme preterm births (≤33 weeks’ gestation) increased from 2.7% in singletons to 11.9% and 57.3% in twins and triplets, respectively.

### Fertility preservation

**Supplementary Figure 4:**
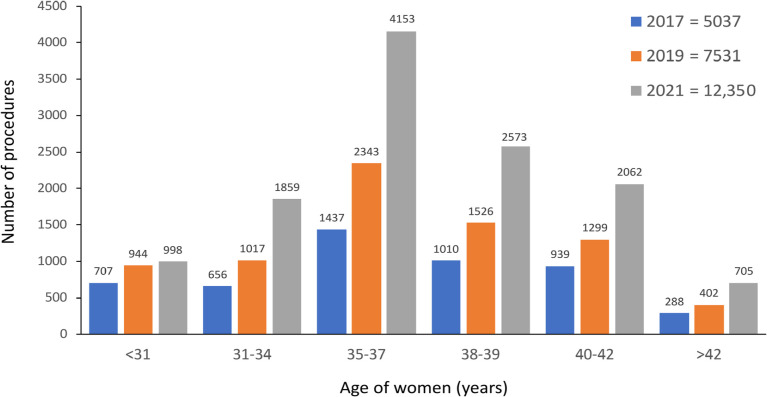
Age of women at fertility preservation in Latin American ART Registry, 2017, 2019, 2021.

In the last 5 years, the number of cycles for fertility preservation has increased almost 2.5 times. In 2021 there were 12,350 initiated cycles of which 11,018 had at least one mature (metaphase II [MII]) oocyte. However, what has not changed is the age distribution of women preserving oocytes (Supplementary Figure 4). Following similar patterns to previous years, almost 77% of women were aged ≥35 years, and 43.2% were ≥38 years. The mean number ± standard deviation of MII oocytes vitrified in women aged ≤34 years was 10.06 (7.6), dropping to 7.59 (6.1) in women aged 35-39 years, and reaching 5.38 (4.1) in women aged ≥40 years. In 61.7% of cases the reason for fertility preservation was fertility postponement, while a cancer diagnosis was the reason in only 3.5% of cases. The rest of the diagnoses (34.8%) included conditions or diseases associated with risks of ovarian insufficiency.

## DISCUSSION

This is the 33rd report on ART procedures performed in Latin America, including 204 certified institutions in 16 countries. Big data have been gathered through a case-by-case data collection system allowing for detailed information and comparisons of outcome and complications among different treatment alternatives. This cycle-based data collection system allows for the detailed stratification of variables influencing reproductive outcome. When examining the information presented here, it is important to consider that, compared with other national registries, this is one of the few multinational cycle-based registries. Therefore, the results reflect the realities of several countries exhibiting different reproductive strategies, including differences such as access to treatment, limitations in the number of embryos transferred and age selection.

The utilization of ART continues to be influenced by the wealth of a country and by the establishment of laws or regulations in favour of building families. When comparing data from 2020 and 2021 there is a continuous growth in Uruguay, which has a law providing universal access to ART, accompanied by a stable economy and a national insurance system which, with few restrictions, covers ART treatments. Uruguay increased utilization from 558 cycles/million in 2020 to 623 cycles/million in 2021. Furthermore, Chile, in the absence of a law regulating ART, established a state programme where two treatments cycles are half-covered by state funds. This increased utilization from 372 in 2020 to 554 cycles/million inhabitants in 2021. On the other hand, the utilization in Argentina, despite a law providing universal care, dropped from 490 to 480 cycles per million inhabitants due to economic restrictions. In 2021, according to national vital statistics by country, 1.43% of births in Uruguay, 1% in Chile and 0.64% in Argentina result from ART treatments. In comparison, the mean ART utilization in European countries for 2019 was 1581 cycles per million, generating between 1.2% and 6.3% of babies born from ART treatments in that year (European IVF Monitoring Consortium (EIM) for the European Society of Human Reproduction and Embryology (ESHRE), 2023).

From a clinical perspective, the question arises of whether the first option should be the transfer of fresh or frozen-thawed embryos. The proportion of FET cycles continues to rise, representing 72.5% of all autologous transfers (see Figure 9). As reported in the past, both the CPR and delivery rate after FET were higher than after fresh transfers, irrespective of the number of embryos transferred. The main reason for this is the higher proportion of blastocyst transfers after FET (89.1%) compared with fresh transfers (55.6%). However, when comparing the elective transfer of fresh blastocysts (Supplementary Table 2) and either FET or freeze-all cycles (Tables 4 and 5), both the CPR and delivery rates were significantly higher after fresh blastocyst transfer (CPR: *p<*0.0001 and *p<*0.0001; delivery rate: *p=*0.0001 and *p<*0.0001, respectively). This suggests that an elective fresh blastocyst transfer should whenever possible be preferred. Overall, when considering the transfer of frozen-thawed embryos, the transfer of embryos after a freeze-all cycle continues to provide better CPR and delivery rate than after regular FET resulting from a failed fresh transfer.

How effective is the transfer of VWO? Most transfers with VWO result from oocyte donation programmes. Therefore, the results obtained after this procedure are an indirect reflection of what should happen after fertility preservation at a young age (as almost all oocyte donors are between 25 and 30 years old). As seen in Supplementary Table 5, the CPR and delivery rate are significantly higher after the transfer of fresh embryos generated from VWO than frozen-thawed embryos derived from VWO (*p=*0.0001 and *p<*0.0001, respectively). Furthermore, not only is the delivery rate lower but so too is the number of multiple births, which indirectly reflects the poorer quality of embryos resulting from a ‘double freezing’, first as oocytes and then as embryos. Therefore, freezing embryos that result from VWO should be left as a rescue procedure when the number of embryos generated exceeds that of those to be transferred. The question arises whether preserving oocytes is equivalent to preserving fertility.

**Supplementary Figure 5:**
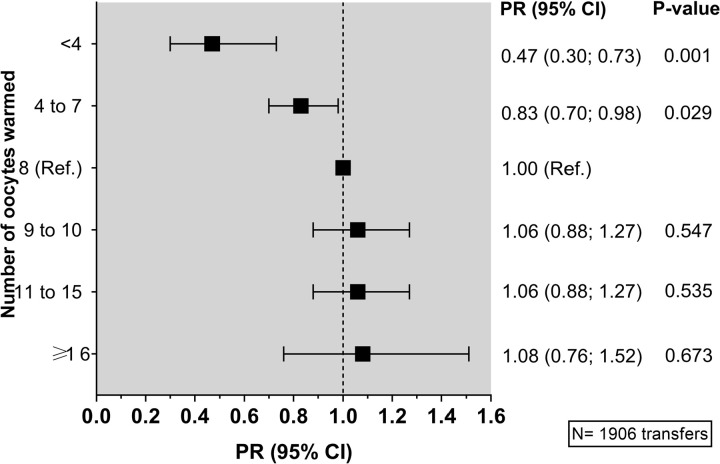
Probability of delivery according to the number of viable vitrified-warmed oocytes.

Preserving reproductive capacity is not equivalent to preserving eggs. As seen in Supplementary Figure 4, 54.5% of women preserving their eggs in Latin America in 2021 were 35-39 years old, and 22.4% were ≥40 years. When preserving eggs, the number of eggs collected and the age of the woman are the most important factors to maintain reproductive capacity at an older age. The data reported here suggest that before deciding to preserve eggs, women need to consider three levels of physiological barrier. The first is the number of vitrified eggs surviving after warming and the chances of normal fertilization after ICSI. The influence of the number of VWO surviving the process is associated with the probability of delivery when transferred as embryos. As seen in Supplementary Figure 5, the probability of giving birth is significantly lower with fewer than eight VWO (in terms of the median number of VWO available in oocyte donors, between four and seven VWO: *p=*0.029; with fewer than four VWO: *p=*0.001). At least for the first transfer (not cumulative), having more than eight oocytes does not seem to improve delivery rates.

The second barrier is the ability to reach the blastocyst stage, and the third barrier is an increasing prevalence of aneuploidy as age progresses. As shown in this report, both blastulation and aneuploidy are strongly influenced by the age of the women (see Figures 7 and 10). In women aged 35-39 years, the chances of zygotes reaching the blastocyst stage is only 35.5%, and 60.1% of those will probably be aneuploid. In women aged ≥40 years, only 28.2% of zygotes will reach the blastocyst stage, and 76.2% of those will be aneuploid. Therefore, if women in these two age groups manage to produce 10 zygotes, which according to the current data is unlikely, the expected number of euploid blastocysts will only be 1.4 and 0.7, respectively.

In contrast to the good reproductive outcome after the transfer of embryos resulting from VWO in young women (Supplementary Table 5), the cryopreservation of oocytes after 35 years and especially after 40 becomes a mere illusion and women should be adequately educated to include these natural barriers in their decision-making process.

In terms of PGT, should it always be performed, and at all ages? The current data related to 5122 homologous embryo transfer cycles that had undergone PGT compared with 23,988 homologous FET cycles without PGT show significant benefits both in increasing the delivery rate and lowering the miscarriage rate in women aged ≥35 years. Many argue that the real measure of effectiveness after PGT should be using follicular aspiration or an initiated cycle as the denominator. The current authors believe that this is not the case since the decision of whether or not to use PGT is only relevant once blastocysts have been formed. In the absence of blastocyst formation, the question of PGT becomes irrelevant.

When there are numerous blastocysts available, there is no doubt about the advantage of PGT. However, a relevant question is whether PGT should be attempted when there is only one blastocyst available for transfer. In the absence of PGT, the delivery rate, during 2021, after the transfer of one non-elective blastocyst (oSET) was 17.6%; however, in a 4-year period, which included 16,975 cases of oSET, the delivery rate fluctuated between 31.7 in women under 32 years and approximately 10% in women beyond 40 years (see Figure 8). In cycles with PGT performed in only one blastocyst in 2021, the delivery rate when an euploid embryo was transferred was 40.9% (786/1921 transfers), but a large proportion (46.5%, 2746/5903) of women with one blastocyst biopsied did not have embryos for transfer due to aneuploidy.

The decision of whether or not to use PGT is further complicated because PGT is not 100% safe for the embryo, nor is it 100% accurate. Some embryos will be damaged during the procedure or wrongly diagnosed, especially in cases of mosaicism, and could have had a chance of implantation if PGT had not been performed. The dilemma involved in this decision includes not only biological considerations, but also social and personal values, which are important to discuss with patients before deciding whether to undertake PGT at all, and especially with only one available embryo.

There is agreement that the cumulative delivery rate or live birth rate is the best marker that patients should consider when deciding whether ART is cost-effective in their case. However, counselling patients is not easy, especially at the cycle start when the number of embryos generated is not yet known. In the cohort of women included in the cumulative delivery rate calculation (12,892), the mean age was 37 (4.32) years and 30% of women were aged ≥40 years. In this cohort, only 33.7% of women had frozen embryos available for a second or third transfer. Therefore, 66.3% of women (8548/12,892) did not have embryos preserved for a second chance and were not exposed to a cumulative probability. This is the reason why the curve representing the cumulative delivery rate is very near the curve representing the delivery rate after fresh transfer. On the other hand, in the subgroup of 4344 women with surplus embryos (mean age 36 [SD 4.08] years), the concept of cumulative delivery rate is more real, and adding frozen to fresh transfers results in a significant improvement (see Figure 12).

## Appendix

**Supplementary Table 1:** List of centers reporting to the Latin American Registry (RLA)

ARGENTINA
• Servicio de Medicina Reproductiva, Instituto Gamma
• Instituto de fertilidad asistida
• Centro de Estudios en Ginecología y Reproducción (CEGYR)
• Centro especializado en reproducción (CER)
• Centro Integral de Ginecología, Obstetricia y Reproducción (CIGOR)
• Centro de Medicina Reproductiva Bariloche , Fertility Patagonia
• Centro de Estudios en Reproducción y Procedimientos de Fertilización Asistida (CRECER)
• FERTILAB
• Fertilis Medicina Reproductiva
• Fertya
• FECUNDART
• Centro de Reproducción, servicio de Ginecología Hospital Italiano
• Mater, Medicina Reproductiva
• Nascentis, Medicina Reproductiva
• HALITUS, Instituto Médico
• PREGNA, Medicina Reproductiva
• Programa de asistencia reproductiva PROAR
• PROCREARTE
• Fertilidad San Isidro
• SARESA, Salud reproductiva Salta
• VITAE, Medicina Reproductiva
BOLIVIA
• CENALFES
• Instituto de Salud Reproductiva (ISARE)
• EMBRIOVID, centro integral de reproducción y especialidades médicas
BRAZIL
• ANDROLAB, Clínica y Laboratorio de Reproducción Humana y Andrología
• ANDROFERT, Centro de Referencia en Reproducción Masculina
• FERTIVITRO, Centro de Reproducción Humana
• BIOS, Centro de Medicina Reproductiva
• FIV-MED
• Centro de Medicina da reproduçao
• VIDA, Centro de Fertilidad
• Clínica FERTWAY
• Nascer-Medicina Reprodutiva Ltda.
• ORIGINARE, Centro de Reproducción Humana
• CLINIFERT, Centro de Reproducción Humana
• CONCEPTUS, Centro de Reproducción Asistida de Ceara
• CONCEBER, Centro de Reproducción Humana
• Clínica Origen
• Clínica Pro-Gerar
• Centro de reproducción humana CONCEPTION
• Centro de Reproducción Humana MONTELEONE
• Centro de reproduçao humana Wahib Hassan
• Fértile Diagnósticos
• CEERH, Centro especializado en Reproducción Humana
• Embrios, centro de reproducción humana
• EMBRYOLIFE, Instituto de Medicina Reproductiva
• CENAFERT, Centro de Medicina Reproductiva
• Instituto VERHUM
• Clínica FERTIBABY BH
• Huntington Brasilia
• FECUNDA, Reproducción Humana
• FELICCITA, Instituto de Fertilidad Ltda.
• HUMANA, Medicina Reproductiva
• FertLiv
• FERTILITY, Centro de Fertilizaçao Asistida
• FERTIL Reproduçao Humana
• REPROFERTY
• FERTICLIN, Clínica de Fertilidad Humana
• FECUNDAR Medicina Reproductiva
Genesis Instituto de reproducción humana de Cascavel PR
GENESIS, Centro de Assistencia en Reproduçao Humana
Genics, medicina reproductiva y genómica FERTIPRAXIS
GERA, Grupo de endoscopia y Reproducción Asistida
• Nucleo de Reproduçao humana do Hospital Moinhos de Vento -GERAR
• Clinica GERAR VIDA
• Cegonha Medicina Reproductiva
• PRIMORDIA, Medicina Reproductiva
• Hospital de Clínicas de Riberao Preto
• HUNTINGTON Campinas
• HUNTINGTON, Centro de Medicina Reproductiva (Sao Paulo)
• JULES WHITE, Centro de Medicina Reproductiva
• HUNTINGTON Vila Mariana
• Ideia Fertil, Santo André
• Ideia Fertil, Sao Paulo
• IMR, Instituto de Medicina Reproductiva e Fetal
• Insemine , Centro de Reproducción Humana
• Centro de Reproducción Humana Santa Johana
• Lab for Life centro de reproduçao humana
• Life reproducción humana
• FERTILITAT, Centro de Medicina Reproductiva
• Clínica Nidus
• Centro de Pesquisa e Reproduçao Humana Nilo Frantz
• Origen, Centro de Medidicina Reproductiva BH
• Procriar, Centro de Medicina Reproductiva y diagnósticos Ltda., Blumenau
• Clínica PRO-CRIAR, Medicina Reproductiva BH
• Clínica PRO NASCER
• Clinica ProSer
• Centro de Reproducción Humana De San Jose de Rio Preto
• Centro de fertilidad Hospital Moinhos de vento
• GENESIS, Centro de Reproducción Humana
• Centro de Reproducción Humana Prof. Franco Junior
• Centro de Ensino y Pesquisa en Reproducción Asistida (CEPRA)
CHILE
• UMR Clínica de la Mujer Antofagasta
• Centro de Estudios Reproductivos (CER)
• Unidad de Medicina Reproductiva, Clínica Alemana
• Unidad de Medicina Reproductiva, Clínica las Condes
• Unidad de Medicina Reproductiva, Clínica de la Mujer
• UMR clínica Indisa
• Programa de Fertilización Asistida I.D.I.M.I.
• Clínica Monteblanco
• Instituto de Medicina Reproductiva Concepción S.A.
• Centro de reproducción humana, Valparaíso
• SG Fertility Chile
COLOMBIA
• Centro FECUNDAR, Cali
• Unidad de fertilidad del Coutry ltda. CONCEPTUM
• Fertility Care Colombia SA
• Centro de fertilidad Clínica de la mujer
• Clínica Eugin
• FERTIVIDA
• Clínica Machicado SAS
• Instituto de Fertilidad Humana S.A.S. (INSER Bogotá)
• IN SER, Instituto Antioqueño de Reproducción (Medellín)
• Novafem SAS
• Procrear
• Profamilia Fertilidad
• Unidad de Fertilidad, Procreación Medicamente Asistida
• Unión temporal IN SER eje cafetero (Pereira)
COSTA RICA
• Azul Fertility expert
ECUADOR
• Clínica INFES
• Clínica de medicina Reproductiva BIOGEPA
• Instituto Nacional de Investigación de Fertilidad y Esterilidad (INNAIFEST)
CONCEBIR, Unidad de Fertilidad y Esterilidad
Centro Ecuatoriano de Reproducción Humana
FERTIMEDEX
Provida Nascer- Clínica Provida
Unidad de Fertilidad Drs. Valdivieso
El SALVADOR
• Latid Fertility Center
GUATEMALA
• Centro de Reproducción Humana S.A. (CER)
• Centro Clínico Gestar (nuevo)
MEXICO
• Biofertility Center
• Centro de Diagnóstico Ginecológico
• Clínica Cerh S e RL de CV
• Dr. Cigüeña
• URA, Unidad de reproducción asistida de Hospital CIMA Hermosillo
• Centro de Cirugía Reproductiva y Ginecología, Unidad de Fertilización In Vitro (REPROGYN)
• Instituto de Innovación Tecnológica y Medicina Reproductiva CITMER (Ciudad de México)
• Centro de Innovación tecnológica y medicina Reproductiva (Monterrey)
• Citmer-Centro de innovación tecnológica y medicina reproductiva Puebla
• Instituto para el estudio de la Concepción Humana IECH
• Centro de Reproducción Asistida del Hospital Español (HISPAREP)
• Centro de Reproducción Asistida de Saltillo
• Creasis
• Centro Universitario de Medicina Reproductiva (CuMER)
• Eligen Fertility center
• Fertilidad Integral
• Fertility Center Cancún
• Fertilita Medicina Reproductiva, Laboratorio in vitro
• Fertygen
• Centro de Medicina reproductiva Filius
• Genesis Centro de Fertilidad (Culiacán)
• Ginecología y Reproducción Asistida GYRA
• Unidad de Medicina Reproductiva del Hospital Ángeles del Pedregal
• IECH de Baja California
• Instituto Mexicano de Alta Tecnología Reproductiva S.C. (INMATER)
• Concibo
• Instituto Médico de la mujer (RED CREA)
• Instituto VIDA Guadalajara-Instituto de Ciencias en Reproducción Humana
• Instituto de Ciencias en Reproducción Humana, VIDA sede Matamoros
• Centro especializado para la atención de la mujer (CEPAM)
• INGENES DF
• INGENES Guadalajara
• Ingenes Monterrey
• Instituto de investigación científica en fertilidad, andrología y reproducción (INICIAR)
• Instituto de Ciencias en Reproducción Humana (VIDA), sede León
• MasFertil
• Instituto de ciencias en reproducción humana del Sureste (Vida Mérida)
• Clínica Nascere
• Centro Origen, Merida
• Plenus, Reproducción Asistida
• PROGEN
• Clínica de Infertilidad y reproducción asistida de Toluca SA de CV
• Centro especializado den esterilidad y reproducción humana
• Unilive
• Instituto de Ciencias en reproducción humana VIDA, ciudad de México.
• Centro CARE
• Vida, Instituto de Reproducción Humana del Noroeste, Tijuana
PANAMA
• IVI Panamá S.A.
• Centro Dr. Camilo Ayllene
• Instituto de salud femenina
• IVF Panamá Centro de reproducción Punta Pacífica (PTY)
PARAGUAY
• Neolife, Medicina y cirugía reproductiva
PERU
Clínica CEFRA, Centro de Fertilidad y Reproducción Asistida
CEFERGIN
CERAS
Centro de Fertilidad y Ginecología del Sur (CFGS)
Clínica de fertilidad del norte, Clinifer de Chiclayo
• Embryofertility
• FERTILAB
• Centro de Fertilidad Germinar
• Inmater, Clinica de fertilidad y reproducción asistida
• Instituto de Reproducción de la Clínica Ricardo Palma
• Clínica Miraflores, Instituto de Ginecología y Fertilidad
• Nacer, centro de reproducción humana de Lima
• NiuVida
• Grupo Pranor San Isidro, Clínica CONCEBIR
• Grupo Pranor, Instituto de Ginecología y Reproducción Monterrico
REPUBLICA DOMINICANA
• Instituto de reproducción y ginecología del Cibao - IREGCI
• Programa de fertilización asistida y medicina perinatal - PROFERT
URUGUAY
• Centro de Esterilidad Montevideo (CEM)
• Centro de Reproducción Humana del Interior
VENEZUELA
• FERTILAB
• Avila Fiv
• Instituto Venezolano de Fertilidad

**Supplementary Table 2:** Clinical pregnancy rate, delivery rate and gestational order in elective and non-elective blastocyst SET and DET in Fresh autologous IVF/ ICSI in 2021.

Type of transfer	Embryo transfers		Clinical pregnancies		Embryo Transfers^*^	Deliveries							
	Number	%	Number	%	Number	Number of deliveries	Delivery rate per embryo transfer^*^ (%)	Singleton (n)	Singleton (%)	Twin (n)	Twin (%)	>Triplets (n)	>Triplets (°/o)
oSET	1,534	43.7	373	24.3	1,503	264	17.6	262	99.2	2	0.8	0	0
eSET	1,978	56.3	808	40.8	1,937	619	32.0	610	98.6	8	1.3	1	0.1
oDET	1,729	49.4	619	35.8	1,666	444	26.6	348	78.4	96	21.6	0	0
eDET	1,768	50.6	880	49.8	1,681	659	39.2	490	74.4	166	25.2	3	0.4

* Excluding cases of pregnancies with loss of follow-up (LFU).

eSET, elective single-embryo transfers; oSET, transfer of only one embryo because there are no more embryos available for transfer; eDET, elective double-embryo transfers; oDET, the transfer of only two embryos because there are no more embryos available for transfer.

**Supplementary Table 3:** Clinical pregnancy rate, delivery rate and gestational order according to the number of fresh embryos transferred in OD in 2021.

Number of embryos transferre	Embryo transfers		Clinical pregnancies		Embryo Transfers^*^	Deliveries							
	Number	%	Number	%	Number	Number of deliveries	Delivery rate per embryo transfer^*^ (%)	Singleton (n)	Singleton (%)	Twin (n)	Twin (°/o)	>Triplets (n)	>Triplets (°/o)
**1**	1,663	41.2	764	45.9	1,620	582	35.9	575	98.8	7	1.2	0	0
**2**	1,997	49.4	1,079	54.0	1,926	856	44.4	598	69.9	252	29.4	6	0.7
**>3**	380	9.4	210	55.2	363	168	46.3	92	54.7	65	38.7	11	6.6
**Total**	4,040	100	2,053	50.8	3,909	1,606	41.1	1,265	78.8	324	20.2	17	1.0

* Excluding cases of pregnancies with loss of follow-up (LFU).

**Supplementary Table 4:** Clinical pregnancy rate, delivery rate and gestational order according to the number of frozen embryos transferred in OD in 2021.

Number of embryos transferre	Embryo transfers		Clinical pregnancies		Embryo Transfers^*^	Deliveries							
	Number	%	Number	%	Number	Number of deliveries	Delivery rate per embryo transfer^*^ (%)	Singleton (n)	Singleton (%)	Twin (n)	Twin (%)	>Triplets (n)	>Triplets (%)
**1**	6,113	60.3	2,529	41.4	5,909	1,875	31.7	1,852	98.8	23	1.2	0	0
**2**	3,455	34.1	1,731	50.1	3,266	1,262	38.6	943	74.8	311	24.6	8	0.6
**>3**	577	5.7	331	57.4	553	253	45.8	160	63.2	85	33.6	8	3.2
**Total**	10,145	100	4,591	45.2	9,728	3,390	34.8	2,955	87.2	419	12.4	16	0.4

* Excluding cases of pregnancies with loss of follow-up (LFU).

**Supplementary Table 5:** Clinical pregnancy rate, delivery rate and gestational order after the transfer of donated frozen warmed oocytes (FWO/OD), 2021.

Number of embryos transferre	Embryo transfers		Clinical pregnancies		Embryo Transfers^*^	Deliveries							
	Number	%	Number	%	Number	Number of deliveries	Delivery rate per embryo transfer^*^ (%)	Singleton (n)	Singleton (°/o)	Twin (n)	Twin (%)	>Triplets (n)	>Triplets (%)
**FWO FRESH**	2033	74.6	1014	49.9	1906	729	38.2	617	84.6	108	14.8	4	0.6
**FWO FET**	694	25.4	286	41.5	636	168	26.4	154	91.7	14	8.3	0	0.0
**TOTAL**	2727	100	1300	47.7	2542	897	35.3	771	86.0	122	13.6	4	0.4

FWO FRESH: Frozen warmed oocytes which have been fertilized and the embryos transferred fresh

FTO FET: Frozen warmed oocytes which have been fertilized and the resulting embryos frozen again before transfer.

* Excluding cases of pregnancies with loss of follow-up (LFU).
